# Development of the standard mouse model for human bacterial vaginosis induced by *Gardnerella vaginalis*

**DOI:** 10.3389/fvets.2023.1226859

**Published:** 2023-09-12

**Authors:** Jinok Kwak, Sriniwas Pandey, Jinho Cho, Minho Song, Eun Sol Kim, Hyunok Doo, Gi Beom Keum, Sumin Ryu, Yejin Choi, Juyoun Kang, Sheena Kim, Jeongyun Kim, Hyeun Bum Kim

**Affiliations:** ^1^Department of Animal Biotechnology, Dankook University, Cheonan, Republic of Korea; ^2^Division of Food and Animal Science, Chungbuk National University, Cheongju, Republic of Korea; ^3^Division of Animal and Dairy Science, Chungnam National University, Daejeon, Republic of Korea; ^4^Department of Physics, Dankook University, Cheonan, Republic of Korea

**Keywords:** *Gardnerella vaginalis*, bacterial vaginosis, mouse model, epithelial exfoliation, cytokine

## Abstract

Bacterial vaginosis (BV) is a polymicrobial syndrome characterized by a diminished number of protective *bacteria* in the vaginal flora. Instead, it is accompanied by a significant increase in facultative and strict anaerobes, including *Gardnerella vaginalis* (*G. vaginalis*). BV is one of the most common gynecological problems experienced by reproductive age-women. Because an ideal and standard animal model for human BV induced by *G. vaginalis* is still underdeveloped, the main objective of this study was to develop a mouse model for human BV induced by *G. vaginalis* to demonstrate the clinical attributes observed in BV patients. A total of 80 female ICR mice were randomly assigned to 4 groups and intravaginally inoculated with different doses of *G. vaginalis*: NC (uninfected negative control), PC1 (inoculated with 1 × 10^5^ CFU of *G. vaginalis*), PC2 (inoculated with 1 × 10^6^ CFU of *G. vaginalis*) and PC3 (inoculated with 1 × 10^7^ CFU of *G. vaginalis*). The myeloperoxidase (MPO) activity and serum concentrations of cytokines (IL-1β, IL-10) in mice administered with *G. vaginalis* were significantly higher than those of the control group. Gross lesion and histopathological analysis of reproductive tract of mice inoculated with *G. vaginalis* showed inflammation and higher epithelial cell exfoliation compared to the control group. In addition, vaginal swabs from the mice inoculated with *G. vaginalis* showed the presence of clue cells, which are a characteristic feature of human BV. Altogether, our results suggested that *G. vaginalis* is sufficient to generate comparable clinical attributes seen in patients with BV.

## 1. Introduction

Under normal condition, vagina harbors mutualistic microflora that is complex, diverse, and dynamic. Vaginal microflora, however, constantly shifts during the menstrual cycle and the entire life of a woman. This ecosystem is mainly composed of bacteria with a predominance of *Lactobacillus* species, along with a few anaerobes and microaerophiles (1, 2). Disruption of this ecosystem provides an opportunity for the pathogens to overgrow and can cause a variety of pathogenic conditions, including bacterial vaginosis (BV) (3–5). BV has become a global concern due to its widespread high prevalence and lack of proper treatment. Although antibiotics are used as treatment, their greatest drawback is high incidence of recurrence within months of treatment (6).

BV is a polymicrobial syndrome characterized by a diminished number of “protective” *Lactobacillus* species (*Lactobacillus crispatus*, *Lactobacillus jensenii*) from vaginal flora, along with a simultaneous significant proliferation of facultative and strict anaerobes, including *Gardnerella vaginalis* (*G. vaginalis*) (7–9). BV frequently affects women of reproductive age, ranging from 15 to 44 years (10). The prevalence of BV varies between nations and between ethnic groups within nations (11). It is interesting to note that this illness is less prevalent in Asia and Europe, while it is most common in several regions of Africa (10). In the United States, BV is more common in non-Caucasian women with 51% African-American, 32% of hispanic women being affected, compared to 23% of Caucasian women (12). According to several studies, numerous sexual behavior-related characteristics, along with other risk factors, have been linked to BV (13). Women are more likely to report BV if they have higher number of sexual partners, are unmarried, and started having sexual intercourse at a young age (10, 14). The clinical signs of symptomatic BV include foul vaginal discharge, vaginal itching, and irritation. However, 50% of the women who fit the diagnostic criteria do not show any symptoms. It is still unclear why some women experience symptoms while others do not (15–17). Regardless of the known etiology of BV, the main causative agent of BV still remains a topic of debate. Though some believe *G. vaginalis* to be the sole causative agent of BV (18), others consider it as one causative agent among the several BV causing bacterial groups (18). One of the main reasons for this confusion is lack of pertinent animal models which can clear up this confusion. Nevertheless, *G. vaginalis* has been widely considered as the main contributor to BV because of its isolation from up to 95% of BV cases (18, 19).

Even though BV often does not cause serious inflammatory conditions (20), several studies have reported increased levels of pro-inflammatory cytokines, such as IL-1β, IL-6, IL-8, and IL-10 in vaginal specimens from women with BV compared to “healthy” controls (21–23). It underscores the need to investigate the inflammatory responses in a well-established mouse model of bacterial vaginosis.

Animal models are an important tool in biomedical research that allows scientists to study human diseases in a controlled environment, test potential treatments, and gain insights into how diseases work (24). However, an ideal and standard animal model for human BV still remains underdeveloped despite the documented health-related problems in patients with BV. Therefore, the aim of this study was to develop a standard mouse model for human BV induced by *G. vaginalis*, which could well describe the clinical attributes seen in patients with BV, and hence help us to better understand the pathogenesis of BV.

## 2. Materials and methods

### 2.1. Preparation of *Gardnerella vaginalis* inoculum

*Gardnerella vaginalis* (KCTC5096) was purchased from Korean Collection for Type Culture (KCTC, Jeongeup, Republic of Korea). *G. vaginalis* is a species of Gram-variable-staining facultative anaerobic bacteria. *G. vaginalis* was cultured in brain heart infusion (BHI) medium (Oxoid, Basingstoke, United Kingdom) supplemented with 10% of fetal bovine serum (FBS) (Gibco, Grand Island, United States) at 37°C for 48 h in 5% CO_2_ atmosphere. *G. vaginalis* concentration was adjusted to 1 × 10^8^ CFU/mL, and serial dilution was performed to prepare concentrations of 1 × 10^5^–10^7^ CFU/mL. For mouse inoculation, the 1 mL of each diluted culture was centrifuged at 5,000 rpm for 10 min, and the resulting pellet was resuspended in 20 μL of sterile phosphate-buffered saline (PBS).

### 2.2. Study design and *Gardnerella vaginalis* inoculation

**Figure 1:**
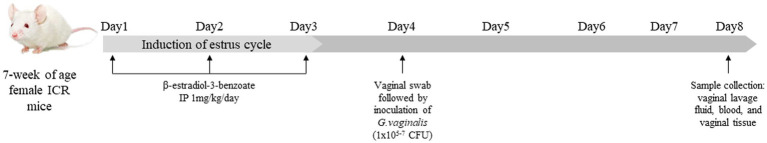
Study design and *Gardnerella vaginalis* inoculation. All mice were intraperitoneally injected with β-estradiol-3-benzoate (1 mg/kg) for 3 days, then PC1, PC2 and PC3 were intravaginally inoculated with 1 × 10^5^ CFU, 1 × 10^6^ CFU and 1 × 10^7^ CFU of *G. vaginalis*, respectively. NC was intravaginally inoculated with PBS instead of *G. vaginalis*.

The animal experimental protocol used in this study was reviewed and approved by the Institutional Animal Care and Use Committee of Dankook University, Cheonan, South Korea (Approval No. DKU-22-028). A total of 80 female ICR mice at 6 weeks of age were purchased from Raonbio Inc. (Yongin, Republic of Korea), and were kept for a week to adapt before the start of experiment. They were fed a commercial rodent diet (Cat No. 2018C, Raonbio Inc., Yongin, Republic of Korea), and housed under controlled environmental conditions: with a temperature (23 ± 1°C), humidity (50 ± 10%) and light (12 h light/12 h dark). The mice were randomly assigned to 4 different groups (20 mice/group): NC, PC1, PC2 and PC3. To establish BV by *G. vaginalis* infection, all mice were intraperitoneally injected with β-estradiol-3-benzoate (1 mg/kg) for 3 days, after then PC1, PC2 and PC3 were intravaginally inoculated with 1 × 10^5^, 1 × 10^6^ and 1 × 10^7^ CFU of *G. vaginalis* in 20 μL of sterile PBS, respectively. The NC group was intravaginally inoculated with 20 μL of sterile PBS without *G. vaginalis* (Figure 1).

On the fourth day of the experiment, before intravaginal inoculation of *G. vaginalis*, vaginal swabs were collected to evaluate the presence of *G. vaginalis* in mice. At the end of the experiment (on the eighth day of the experiment), vaginal lavage fluid (VLF), blood samples, and vagina were collected for the analyses. Briefly, VLF was collected after anesthetizing the mice with avertin (2,2,2-Tribromoethanol, 240 mg/kg) (SigmaAldrich, St. Louis, United States) through the intraperitoneal injection (IP) (Figure 1). VLF was collected by gently flushing the vagina with 50 μL of sterile PBS using a pipette. The collected VLF was used to confirm *G. vaginalis* infection in mice. Blood was also collected from the facial vein in a 1.5 mL Eppendorf tube and left for 30 min at room temperature. Then it was centrifuged for 15 min at 4,000 rpm to separate serum which was used to measure serum concentrations of cytokines. The mice were then sacrificed by cervical dislocation to collect vagina for myeloperoxidase (MPO) activity assay and hematoxylin and eosin (H&E) staining.

### 2.3. Prescreening and reisolation of *Gardnerella vaginalis*

To prescreen for *G. vaginalis* in mice prior to the experiment, the vaginal swabs were taken and spread on Columbia Blood agar (Kisanbio Inc., Seoul, Republic of Korea) (Columbia Agar 43 g/L, defibrinated sheep blood 5%) with *Gardnerella* supplements (gentamycin 0.002 g, nalidixic acid 0.015 g, amphotericin B 0.0001 g/vial/500 mL) (Kisanbio Inc., Seoul, Republic of Korea). Briefly, the swab tip was rolled on the entire agar surface area, then the spreader was used to spread the sample evenly over the entire surface of the agar plate. The plates were incubated at 37°C for 48 h in 5% CO_2_ atmosphere.

For *G. vaginalis* isolation from the inoculated mice, the collected VLF was 10^5^ folds diluted in a sterile PBS, then 10 μL of diluted VLF was spread onto Columbia Blood Agar (Kisanbio Inc., Seoul, Republic of Korea) (Columbia Agar 43 g/L, defibrinated sheep blood 5%) with *Gardnerella* supplements (gentamycin 0.002 g, nalidixic acid 0.015 g, amphotericin B 0.0001 g/vial/500 mL) (Kisanbio Inc., Seoul, Republic of Korea). The agar plates were then incubated at 37°C for 48 h in 5% CO2 atmosphere for isolation of *G. vaginalis* in order to evaluate *G. vaginalis* infection. Colonies were counted and reported as restored colony forming units (CFU)/mL of *G. vaginalis.* To confirm the identity of the isolates from vaginal fluid samples of inoculated mice as the same *G. vaginalis* strain (KCTC5096) used for inoculation, we conducted the *16S rRNA* gene sequencing using universal primers: 27F (5′-AGAGTTTGATCCTGGCTCAG-3′) and 1492R (5′-TACGGYTACCTTGTTACGACTT-3′). The *16S rRNA* gene sequences of *G. vaginalis* strain (KCTC5096) used for inoculation were compared to those of isolates from vaginal fluid samples of inoculated mice using the Molecular Evolutionary Genetics Analysis 11 software (Biodesign Institute, AZ, United States) and NCBI nucleotide blast. Additionally, Gram staining of the isolates from vaginal fluid samples of inoculated mice was conducted to confirm the identity of the isolates as *G. vaginalis*. The stained slides were observed under oil immersion field (1,000×) using a light microscope.

### 2.4. Gram staining of vaginal smears

Vaginal swabs were collected from the mice before *G. vaginalis* inoculation and 4 days post-inoculation. The vaginal swabs were gently smeared and dried on a glass slide, heat-fixed, and then stained using a BD BBL^™^ Gram Stain Kit (Becton, Dickinson and Company, NJ, United States). The stained slides were observed under oil immersion field (1,000×) using a light microscope.

### 2.5. Assessment of myeloperoxidase activity in vaginal tissue

The assessment of MPO activity was performed using Myeloperoxidases Colorimetric Activity Assay Kit (Sigma-Aldrich, St. Louis, United States). Briefly, vaginal tissue was rapidly homogenized in 4 volumes of MPO assay buffer and centrifuged for 10 min at 13,000 × g at 4°C to remove insoluble material. Then, the 50ul of supernatant of homogenate was loaded into 96 well plate, and the MPO activity assay was carried out following the manufacturer’s instructions. The absorbance was measured at 412 nm.

### 2.6. Assessment of serum concentrations of IL-1β and IL-10

The concentrations of IL-1β and IL-10 in mouse serum were measured using Quantikine ELISA Kit (R&D Systems, Minneapolis, United States) following the manufacturer’s instructions. For IL-1β, collected serum was used without dilution, and both the standard and sample were assayed in duplicate. For IL-10, serum was 2 folds diluted with Calibrator Diluent RD5T of Quantikine ELISA Kit (R&D Systems, Minneapolis, United States), and the sample were assayed in duplicate. The absorbance was measured at 450 nm.

### 2.7. Gross lesion and histopathological examination of vagina

The gross lesion of the excised reproductive tract (vagina and uterine horns) of mice inoculated with *G. vaginalis* was evaluated. The histological alterations in vaginal tissues in form of epithelial exfoliation were evaluated through H&E staining. For H&E staining, the vaginal tissue located 5 mm below the cervix was excised and gently washed in PBS to remove blood. Subsequently, vaginal tissues were fixed in 10% formalin for 24 h at room temperature, followed by placing in paraffin. H&E staining was performed by the K2O Co. (Siheung, Republic of Korea). The obtained slides were observed in Olympus CKX53 (Olympus, Tokyo, Japan) to evaluate the degree of epithelial exfoliation. The ZEN3.4 software (Carl Zeiss AG, Oberkochen, Germany) was utilized for the quantitative analysis of the thickness of keratinized stratified squamous epithelium, using the slide scan files provided by K2O Co. (Siheung, Republic of Korea). Three different areas of the keratinized stratified squamous epithelium were randomly measured in each mouse vagina using the ZEN3.4 software. For the quantitative analysis of the reproductive tract length, the distance from the ovary to the cervix was measured using ImageJ software (National Institutes of Health, MD, United States), and subsequently utilized for the statistical analysis.

### 2.8. Statistical analysis

The value from each individual animal was measured and used for the statistical analysis. All the presented statistical analysis was conducted using GraphPad Prism 8.0 software (GraphPad Software, Inc., San Diego, United States). Significant differences in values between groups was determined based on ANOVA, and the level of statistical significance was set at *p* < 0.05. Dunnett’s multiple comparisons test was used for the statistical evaluation of differences between the control (NC) and the infection groups (PC1, PC2, PC3).

## 3. Results

### 3.1. Prescreening and reisolation of *Gardnerella vaginalis*

No *G. vaginalis* was isolated from the mice before intravaginal inoculation with *G. vaginalis*. However, the *G. vaginalis* infection in mice was confirmed by counting the colony forming units (CFU) in VLF collected on 4 days post-inoculation (dpi). As shown in Table 1, PC3 showed the highest bacterial count (183.16 ± 26.77 × 10^5^ CFU/mL), which was expected due to inoculation with the highest dose of GV (1 × 10^7^ CFU). The CFU counts in PC1 and PC2 were (83.3 ± 20.83) × 10^5^ CFU/mL and (111.2 ± 18.95) × 10^5^ CFU/mL, respectively. The bacterial colonies from the mice inoculated with *G. vaginalis* were confirmed to be *G. vaginalis* (KCTC5096) through the *16S rRNA* gene sequencing analysis. When we compared the sequences of these two strains [*G. vaginalis* strain (KCTC5096) used for inoculation and the isolates from vaginal fluid samples of inoculated mice], we confirmed that they were exactly the same, showing a 100% match (Supplementary Figures S1A,B). Furthermore, Gram staining of the isolates from vaginal fluid samples of inoculated mice revealed Gram-variable-staining bacteria, confirming the identity of the isolates as *G. vaginalis* (Supplementary Figures S1C,D).

**Table 1:** The colony counts of *Gardnerella vaginalis* isolated from vaginal lavage fluid (VLF) on 4 days post-inoculation.

Treatment group	Inoculation dose	CFU/mL^a^
NC	—	—
PC1	1 × 10^5^ CFU/mouse	83.3 ± 20.83 × 10^5^
PC2	1 × 10^6^ CFU/mouse	111.2 ± 18.95 × 10^5^
PC3	1 × 10^7^ CFU/mouse	183.16 ± 26.77 × 10^5^

NC, negative control; PC1, positive control inoculated with 1 × 10^5^ CFU of *G. vaginalis* per mouse; PC2: positive control inoculated with 1 × 10^6^ CFU of *G. vaginalis* per mouse; PC3, positive control inoculated with 1 × 10^7^ CFU of *G. vaginalis* per mouse.

a CFU/mL was presented as means ± SD × 10^5^.

### 3.2. Gram staining of vaginal smears

**Figure 2:**
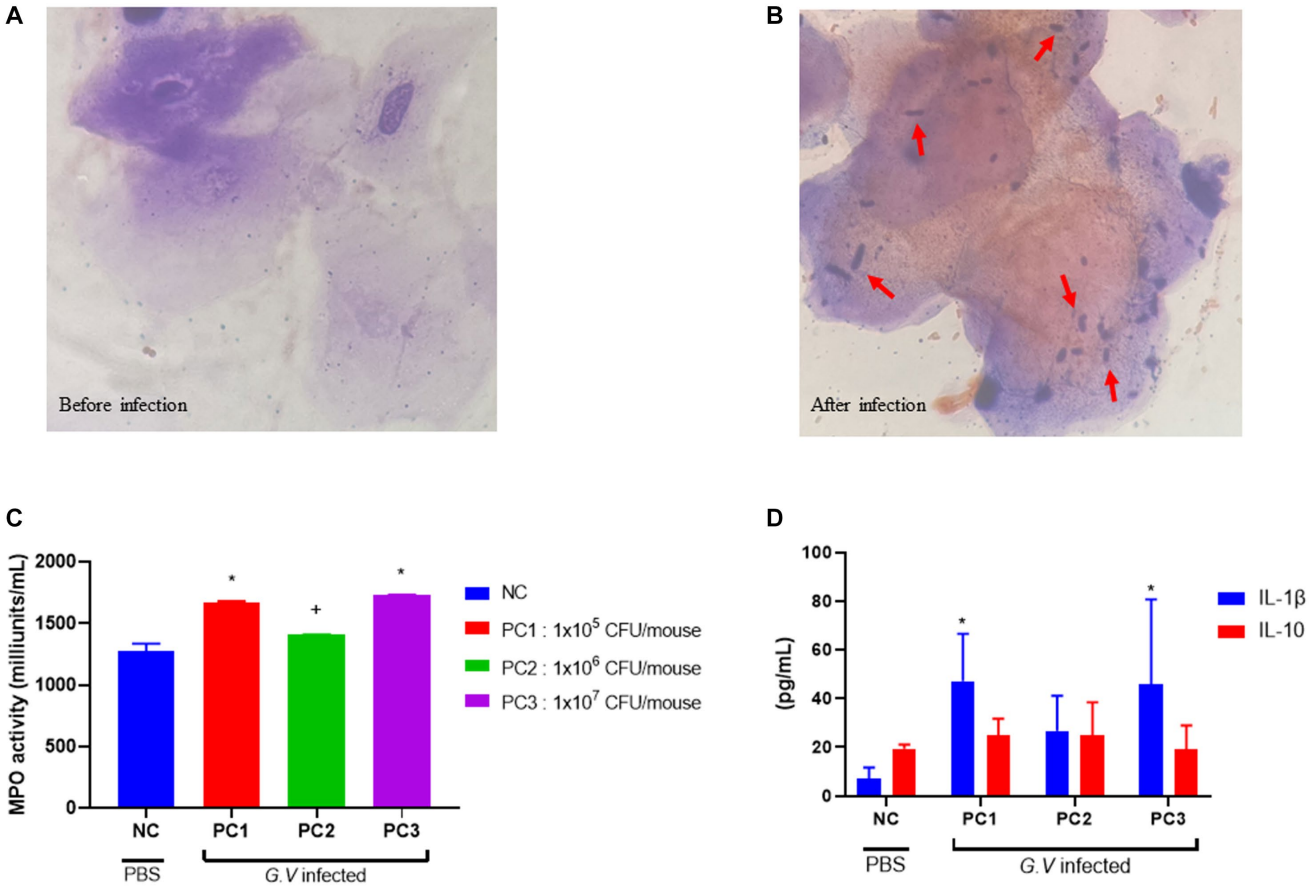
Gram staining of vaginal smears **(A,B)**, assessment of MPO activity in vaginal tissue **(C)**, and serum concentration of IL-1β and IL-10 **(D)**. **(A)** Gram-stained smears of vaginal fluid (before *G. vaginalis* inoculation). No bacterium were detected in the epithelial cells of the mouse vagina prior to *G. vaginalis* inoculation (1000× magnification). **(B)** Gram-stained smears of vaginal fluid (4 days after *G. vaginalis* inoculation). The presence of clue cells, characterized by vaginal epithelial cells covered in bacteria, was confirmed in the vaginal smear of infected mice. The red arrows indicate Gram-variable-staining *G. vaginalis* (1000× magnification). **(C)** Assessment of MPO activity in vaginal tissue. **(D)** Serum concentration of IL-1β and IL-10. The error bars in the graph represent the calculated standard deviation. The significance level of *p* < 0.05 was denoted as “*”, indicating a statistically significant difference. The symbol “+” indicates no statistically significant difference but rather a tendency. The value from each individual animal was measured and used for the statistical analysis (20 mice/group). Significant differences in values between groups was determined based on ANOVA. Dunnett’s multiple comparisons test was used for the statistical evaluation of differences between the control (NC) and the infection groups (PC1, PC2, PC3). NC, negative control; PC1, positive control inoculated with 1 × 10^5^ CFU of *G. vaginalis*; PC2, positive control inoculated with 1 × 10^6^ CFU of *G. vaginalis*; PC3, positive control inoculated with 1 × 10^7^ CFU of *G. vaginalis*.

Histopathologically, BV is diagnosed by presence of ‘clue’ cells, which are essentially vaginal epithelial cells covered in bacteria (25). No bacterium were detected in the epithelial cells of the mouse vagina before *G. vaginalis* inoculation (Figure 2A). However, the results from gram-staining of vaginal epithelial cells on slides prepared from mouse vaginal swabs indicated the presence of clue cells in the infected mouse vaginal smear, which is a characteristic feature of human BV (Figure 2B).

### 3.3. MPO activity in vaginal tissue

MPO is a lysosomal protein abundantly produced in neutrophils that participates in neutralization bacteria and other microorganisms upon activation of neutrophils (4). MPO activity was assessed as a biochemical index reflecting the degree of neutrophil infiltration in vaginal tissue harvested from mice infected with *G. vaginalis*. Vaginal tissue lysate was used to assay the MPO activity. The highest MPO activity was observed in PC3 (1,731 ± 2 milliunits/mL), followed by PC1 (1,688 ± 8 milliunits/mL) and PC2 (1,408 ± 2 milliunits/mL), respectively (Figure 2C). MPO activity was lowest in the negative control (NC) group (1,277 ± 47 milliunits/mL). The MPO activities in PC1 and PC3 were significantly different (*p* < 0.05) from NC group. The MPO activities in PC2 was higher than that of NC, but there was no statistically significant difference.

### 3.4. Expression of pro & anti-inflammatory cytokines

The levels of inflammatory cytokines were measured in the serum of BV-induced mice. The level of IL-1β and IL-10 was highest in PC1 (47.23 ± 8.68 pg/mL, 24.76 ± 3.49 pg/mL), followed by PC3 (45.75 ± 15.68 pg/mL, 19.23 ± 4.35 pg/mL) and PC2 (26.71 ± 7.21 pg/mL, 24.90 ± 6.78 pg/mL), respectively (Figure 2D). The serum levels of IL-1β and IL-10 tended to be higher in mice (PC1, PC2, and PC3) inoculated with *G. vaginalis* compared to the control group (NC).

### 3.5. Gross lesions and histopathological examination of female reproductive tract

**Figure 3:**
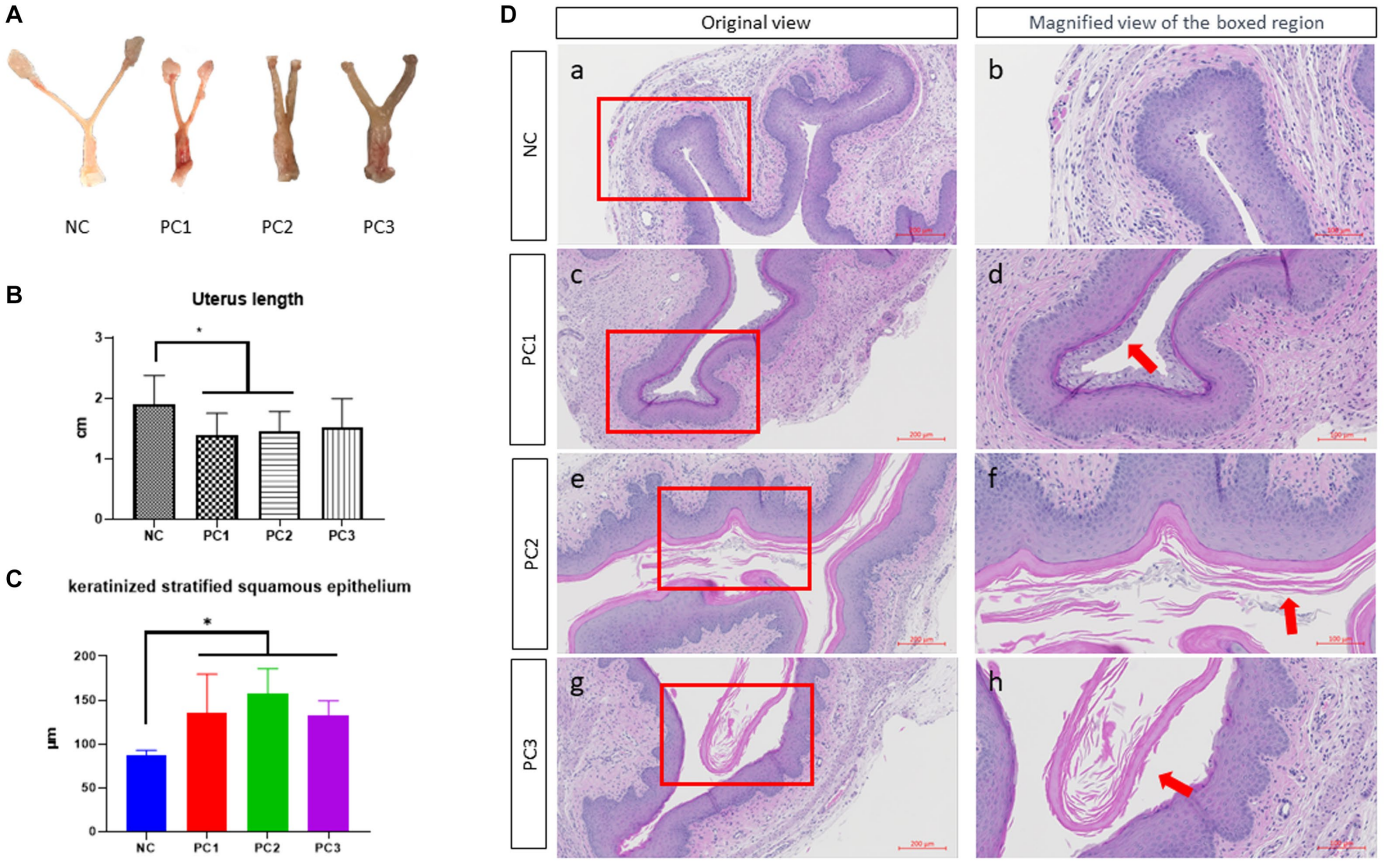
Gross lesion and histopathological examination of vagina. **(A)** The gross lesion of the excised reproductive tract (vagina and uterine horns) of mice. **(B)** The quantitative analysis of the reproductive tract length. For the quantitative analysis of the reproductive tract length, the distance from the ovary to the cervix was measured using ImageJ software (National Institutes of Health, MD, United States), and subsequently utilized for the statistical analysis. **(C)** Quantitative analysis of the thickness of keratinized stratified squamous epithelium. For the quantitative analysis of the thickness of keratinized stratified squamous epithelium, three different areas of the keratinized stratified squamous epithelium were randomly measured in each mouse vagina using the ZEN3.4 software (Carl Zeiss AG, Oberkochen, Germany). **(D)** Images of the hematoxylin and eosin stained (H&E) stained vaginal tissue sections. Scale bars located in the lower right-hand corner of each figure. The red arrows indicate the exfoliated epithelial cells. NC, negative control; PC1, positive control inoculated with 1 × 10^5^ CFU of *G. vaginalis*; PC2, positive control inoculated with 1 × 10^6^ CFU of *G. vaginalis*; PC3, positive control inoculated with 1 × 10^7^ CFU of *G. vaginalis*.

The reproductive tracts of BV-induced female mice were depicted in Figure 3A. The overall thickness of the reproductive tract in BV-induced mice was increased compared to the control group (NC), and was thickest in PC3, followed by PC2 and PC1. The control group (NC) showed a nomal structure of the uterus and vagina. The abnormal thickness observed in treatment groups may be attributed to edema, a classical sign of inflammation in mice infected with *G. vaginalis*. The vagina of BV-induced female mice was comparatively more swollen than NC, and the swelling extended to the uterine horns as well (Figure 3A). In BV-induced mice, the overall length of the uterus exhibited a significant decrease compared to the control group (NC). The lengths were as follows: NC (1.905 cm ± 0.149 cm), PC1 (1.388 cm ± 0.115 cm), PC2 (1.451 cm ± 0.104 cm), and PC3 (1.508 cm ± 0.171 cm) (Figure 3B). The harvested vaginal tissue was stained with H&E staining to confirm the alterations present in the tissue. The quantitative analysis of keratinized stratified squamous epithelium thickness revealed a significant increase in the control group compared to the treatment groups as follows: NC (86.988 μm ± 2.610 μm), PC1 (134.88 μm ± 19.993 μm), PC2 (156.967 μm ± 12.961 μm), and PC3 (133.125 μm ± 7.244 μm) (Figure 3C). In addition, the treatment group (PC3) exhibited significantly higher epithelial cell exfoliation and an increase in epithelial thickness than negative control (NC). The other two treatment groups (PC1 and PC2) also showed considerably more epithelial exfoliation than the control group (Figure 3D).

## 4. Discussion

In the present study, we showed that *G. vaginalis* was sufficient to generate comparable BV in a mice model as those seen in patients with BV. Three different doses of *G. vaginalis* suspension (1 × 10^5^ CFU, 1 × 10^6^ CFU and 1 × 10^7^ CFU) were sufficient to induce BV in mice. The establishment of BV in mice was confirmed by various test results including the gross examination of genital tract, and histopathological study of the excised vaginal tissue.

MPO activity can be used as a biochemical marker to reflect the level of polymorphonuclear cell accumulation in the vaginal tissue of mice. Previous studies have shown increased MPO activity in mice with infection (4, 26, 27) compared to non-treated normal mice. Our results also showed elevated levels of MPO activity in all the three groups infected with *G. vaginalis* compared to the negative control group (Figure 2C). Furthermore, the values of group PC1 and PC3 were significantly different from those of the NC group (*p* < 0.05).

Although *G. vaginalis* is present on the surface of vagina tissue, some patients do not exhibit obvious signs of inflammation such as redness and swelling, which contradicts with the studies reporting increased levels of inflammatory cytokines (28–30). In our study, we observed that the concentration of IL-1β was higher in mice inoculated with *G. vaginalis* (PC1, PC2, PC3) than in the negative control mice (NC) (Figure 2D). IL-1β is a pro-inflammatory cytokine produced by various immune cells in response to infection and injury in order to provide resistance to pathogens (31). Therefore, our result indicates that *G. vaginalis* induced an inflammatory response in the vaginal tissues of mice. This observation is consistent with previous studies that have reported elevated levels of IL-1β in patients with BV. Hedge et al. measured the cytokine level in the serum and vaginal wash of patients with BV (30, 32) They found that IL-1β was significantly higher (*p* < 0.05) in vaginal washes of patients with BV than in normal women. In addition, women with BV who had douched and women with BV but had not douched had high level of IL-1β than the ones who did not have BV (29). Vaginal douching is an act of washing the vagina with a liquid solution to maintain personal hygiene or for cosmetic purposes, to treat an infection, to clean up after menstruation or sexual intercourse (33). Elevated serum concentrations of IL-1β from this study also correlated with findings from a separate study that reported high levels of IL-1β associated with the initiation of an antigen-specific mucosal immune response in women with BV (21).

IL-10, an anti-inflammatory cytokine, was found to be higher in the mice inoculated with *G. vaginalis* (treatment groups PC1, PC2 and PC3) than the control group (NC) (Figure 2D). Sierra et al. also found elevated levels of IL-10 in the cervicovaginal fluid of mice infected with *G. vaginalis* (34). But in another study, lower level of IL-10 was measured in mice infected with *G. vaginalis* than the mice infected with *G. vaginalis* but also administered with *Lactobacillus* (35). The study by Jang et al. (27) suggested that the increase in IL-10 expression observed in mice inoculated with *G. vaginalis* and administered with lactic acid bacteria might be due to a systemic immunomodulatory effect. While certain blood cytokine measurements (such as IL-10) did not demonstrate statistical significance, it is crucial to consider the overall findings of the study when interpreting the observed results. In addition to cytokine measurements, a tissue MPO assay revealed a significant increase in MPO levels in the infection group. This finding highlighted heightened neutrophil activity and inflammation in the vaginal tissues of the infected animals. The increased serum levels of IL-1β, coupled with high MPO levels, strongly suggested that immune responses and local inflammation remained consistent in the infected animals. The examination of inflammatory responses in this model has long been emphasized as a necessary area of research, and we believe that our study holds value as an investigation into bacterial vaginal diseases.

The fact that *G. vaginalis* induced inflammation was also supported by the condition of the genital tract in our study. The genital tracts (vagina and uterine horns) of treatment groups were swollen and showed signs of edema caused by inflammation (Figure 3A). The signs of swelling in the genital tracts of treatment groups (PC1, 2, and 3) were distinguished in comparison to the negative control (NC) which had normal thickness and structure. The swelling increased in the treatment groups in the order of doses given, maximum thickness was seen in the treatment group inoculated with 1 × 10^7^ CFU of *G. vaginalis*. Inflammatory lesions were also observed in the vagina of gnotobiotic mice infected with *G. vaginalis* (36). Although BV is considered as a vaginal illness, many studies have demonstrated that bacterial infections in the vagina can also move to the upper genital tract (37–39), which is supported by the swelling of uterine horns in the mice of treatment groups in comparison to the negative control in this study. Overall, the expression of pro and anti-inflammatory cytokines, along with the swelling of the genital tract clearly suggested that *G. vaginalis* caused the pathologic changes in the mouse genital tracts.

We observed that the Gram staining of mouse vaginal smears revealed the presence of clue cells, a characteristic feature often associated with bacterial vaginosis (BV) in humans (Figure 2B). This finding aligns with previous reports in human studies, where clue cells have been identified as a key diagnostic marker for BV (25). Consistent with our observations, a study that utilized *G. vaginalis* strains to induce mouse vaginosis reported the presence of epithelial cells with attached bacteria, reminiscent of clue cells (40).

In Figures 3A,B, we noticed a trend of decreased uterus length in the infection group compared to the control group. PC1 and PC2 exhibited significant differences compared to NC (*p* < 0.05). This trend could potentially be attributed to the influence of estradiol on uterine contractility, which might be exacerbated by *G. vaginalis* infection (41). Further investigations are required to establish a direct causal relationship between *G. vaginalis* infection, estradiol, and uterine muscle contraction.

Vaginal epithelial cell exfoliation is regarded as a key clinical characteristic in patients with BV (42). While exfoliation is a mechanism of protection by removing adhered bacteria, excessive exfoliation can expose underlying tissue, providing an opportunity for other BV-related bacteria to infect and to increase the risks of secondary infection. In the present study, exfoliation was clearly observed in treatment group (PC3) while it was reduced in PC1 and PC2. It was completely absent in vaginal tissue of mice of control group (Figure 3D). In a mouse model designed by Gilbert et al. (40), epithelial exfoliation was also observed in the vaginal tissue of the mice infected with *G. vaginalis*. Altogether, the results from our current study provide substantial evidence suggesting *G. vaginalis* can actively contribute to the development of BV-related clinical characteristics.

## 5. Conclusion

Overall, the results of this study revealed that *G. vaginalis* alone was sufficient to generate the clinical characteristics similar to those seen in patients with BV. Therefore, this mouse model can bring us one step closer to understanding the persistence of BV and its related problems in females. The standard mouse model for human bacterial vaginosis induced by *G. vaginalis* will help us to better understand the pathophysiology of BV.

## Data availability statement

The raw data supporting the conclusions of this article will be made available by the authors, without undue reservation.

## Ethics statement

The animal study was approved by Animal Care and Use Committee of Dankook University, Cheonan, South Korea (Approval No. DKU-22-028). The study was conducted in accordance with the local legislation and institutional requirements.

## Author contributions

HK, MS, JC, and JeK contributed to conception and design of the study. JiK, EK, HD, GK, SR, YC, and JuK performed the experiments. SP wrote the first draft of the manuscript. JiK, JC, and MS wrote section of the manuscript. JiK, EK, and GK organized the database. JiK, HD, and SK performed the statistical analysis. HK review and editing last manuscript. All authors contributed to the article and approved the submitted version.

## Funding

This research was supported by the Bio & Medical Technology Development Program of the National Research Foundation (NRF) & funded by the Korean Government (MSIT) (No. NRF-2022M3A9I5082342).

## Conflict of interest

The authors declare that the research was conducted in the absence of any commercial or financial relationships that could be construed as a potential conflict of interest.

## Publisher’s note

All claims expressed in this article are solely those of the authors and do not necessarily represent those of their affiliated organizations, or those of the publisher, the editors and the reviewers. Any product that may be evaluated in this article, or claim that may be made by its manufacturer, is not guaranteed or endorsed by the publisher.

## Abbreviations

BV: Bacterial vaginosis
Dpi: Day post inoculation
VLF: Vaginal lavage fluid
MPO: Myeloperoxidase activity
IP: Intraperitoneal injection
CFU: Colony forming unit
H&E: Hematoxylin and eosin

## References

[1] Chen X Lu Y Chen T Li R. The female vaginal microbiome in health and bacterial vaginosis. Front Cell Infect Microbiol. (2021) 11:631972. doi: 10.3389/fcimb.2021.807462, PMID: 33898328 PMC8058480

[2] Danielsson D Teigen PK Moi H. The genital econiche: focus on microbiota and bacterial vaginosis. Ann N Y Acad Sci. (2011) 1230:48–58. doi: 10.1111/j.1749-6632.2011.06041.x, PMID: 21824165

[3] Chee WJY Chew SY Than LTL. Vaginal microbiota and the potential of Lactobacillus derivatives in maintaining vaginal health. Microb Cell Factories. (2020) 19:203. doi: 10.1186/s12934-020-01464-4, PMID: 33160356 PMC7648308

[4] Choi SI Won G Kim Y Kang CH Kim GH. Lactobacilli strain mixture alleviates bacterial vaginosis through antibacterial and antagonistic activity in *Gardnerella vaginalis*-infected C57BL/6 mice. Microorganisms. (2022) 10:471. doi: 10.3390/microorganisms10020471, PMID: 35208925 PMC8880492

[5] Paladine HL Desai UA. Vaginitis: diagnosis and treatment. Am Fam Physician. (2018) 97:321–9. PMID: 29671516

[6] Wu S Hugerth LW Schuppe-Koistinen I Du J. The right bug in the right place: opportunities for bacterial vaginosis treatment. NPJ Biofilms Microbiomes. (2022) 8:34. doi: 10.1038/s41522-022-00295-y, PMID: 35501321 PMC9061781

[7] Pybus V Onderdonk AB. Microbial interactions in the vaginal ecosystem, with emphasis on the pathogenesis of bacterial vaginosis. Microbes Infect. (1999) 1:285–92. doi: 10.1016/S1286-4579(99)80024-0, PMID: 10602662

[8] Randis TM Ratner AJ. *Gardnerella* and *Prevotella*: co-conspirators in the pathogenesis of bacterial vaginosis. J Infect Dis. (2019) 220:1085–8. doi: 10.1093/infdis/jiy705, PMID: 30715397 PMC6736359

[9] Abou Chacra L Fenollar F Diop K. Bacterial vaginosis: what do we currently know? Front Cell Infect Microbiol. (2022) 11:672429. doi: 10.3389/fcimb.2021.672429 35118003 PMC8805710

[10] Javed A Parvaiz F Manzoor S. Bacterial vaginosis: an insight into the prevalence, alternative treatments regimen and it’s associated resistance patterns. Microb Pathog. (2019) 127:21–30. doi: 10.1016/j.micpath.2018.11.046, PMID: 30502515

[11] Kenyon C Colebunders R Crucitti T. The global epidemiology of bacterial vaginosis: a systematic review. Am J Obstet Gynecol. (2013) 209:505–23. doi: 10.1016/j.ajog.2013.05.006, PMID: 23659989

[12] Bautista CT Wurapa E Sateren WB Morris S Hollingsworth B Sanchez JL. Bacterial vaginosis: a synthesis of the literature on etiology, prevalence, risk factors, and relationship with chlamydia and gonorrhea infections. Mil Med Res. (2016) 3:4. doi: 10.1186/s40779-016-0074-5, PMID: 26877884 PMC4752809

[13] Verstraelen H Verhelst R Vaneechoutte M Temmerman M. The epidemiology of bacterial vaginosis in relation to sexual behaviour. BMC Infect Dis. (2010) 10:81. doi: 10.1186/1471-2334-10-81, PMID: 20353563 PMC3161362

[14] Schwebke JR Desmond R. Risk factors for bacterial vaginosis in women at high risk for sexually transmitted diseases. Sex Transm Dis. (2005) 32:654–8. doi: 10.1097/01.olq.0000175396.10304.62, PMID: 16254538

[15] Hay P. Bacterial vaginosis. Medicine. (2014) 42:359–63. doi: 10.1016/j.mpmed.2014.04.011

[16] Bradshaw CS Sobel JD. Current treatment of bacterial vaginosis-limitations and need for innovation. J Infect Dis. (2016) 214:S14–20. doi: 10.1093/infdis/jiw159, PMID: 27449869 PMC4957510

[17] Schwebke JR. Asymptomatic bacterial vaginosis: response to therapy. Am J Obstet Gynecol. (2000) 183:1434–9. doi: 10.1067/mob.2000.107735, PMID: 11120507

[18] Schwebke JR Muzny CA Josey WE. Role of *Gardnerella vaginalis* in the pathogenesis of bacterial vaginosis: a conceptual model. J Infect Dis. (2014) 210:338–43. doi: 10.1093/infdis/jiu089, PMID: 24511102

[19] Hickey RJ Forney LJ. *Gardnerella vaginalis* does not always cause bacterial vaginosis. J Infect Dis. (2014) 210:1682–3. doi: 10.1093/infdis/jiu303, PMID: 24855684 PMC4334793

[20] Mitchell C Marrazzo J. Bacterial vaginosis and the cervicovaginal immune response. Am J Reprod Immunol. (2014) 71:555–63. doi: 10.1111/aji.12264, PMID: 24832618 PMC4128638

[21] Cauci S Driussi S Guaschino S Isola M Quadrifoglio F. Correlation of local interleukin-1beta levels with specific IgA response against *Gardnerella vaginalis* cytolysin in women with bacterial vaginosis. Am J Reprod Immunol. (2002) 47:257–64. doi: 10.1034/j.1600-0897.2002.01096.x, PMID: 12148539

[22] Cauci S Culhane JF Di Santolo M McCollum K. Among pregnant women with bacterial vaginosis, the hydrolytic enzymes sialidase and prolidase are positively associated with interleukin-1β. Am J Obstet Gynecol. (2008) 198:132.e1–7. doi: 10.1016/j.ajog.2007.05.035, PMID: 17714681

[23] Herbst-Kralovetz MM Pyles RB Ratner AJ Sycuro LK Mitchell C. New systems for studying intercellular interactions in bacterial vaginosis. J Infect Dis. (2016) 214:S6–S13. doi: 10.1093/infdis/jiw130, PMID: 27449872 PMC4957508

[24] Franco NH. Animal experiments in biomedical research: a historical perspective. Animals. (2013) 3:238–73. doi: 10.3390/ani3010238, PMID: 26487317 PMC4495509

[25] Amegashie CP Gilbert NM Peipert JF Allsworth JE Lewis WG Lewis AL. Relationship between Nugent score and vaginal epithelial exfoliation. PLoS One. (2017) 12:e0177797. doi: 10.1371/journal.pone.0177797, PMID: 28562623 PMC5451030

[26] Lanza M Scuderi SA Capra AP Casili G Filippone A Campolo M. Effect of a combination of pea protein, grape seed extract and lactic acid in an in vivo model of bacterial vaginosis. Sci Rep. (2023) 13:2849. doi: 10.1038/s41598-023-28957-7, PMID: 36807330 PMC9938223

[27] Jang SE Jeong JJ Choi SY Kim H Han MJ Kim DH. *Lactobacillus rhamnosus* HN001 and *Lactobacillus acidophilus* La-14 attenuate *Gardnerella vaginalis*-infected bacterial vaginosis in mice. Nutrients. (2017) 9:531. doi: 10.3390/nu9060531, PMID: 28545241 PMC5490510

[28] Hemalatha R Ramalaxmi BA KrishnaSwetha G Kumar PU Madusudhan D Balakrishna N. Cervicovaginal inflammatory cytokines and sphingomyelinase in women with and without bacterial vaginosis. Am J Med Sci. (2012) 344:35–9. doi: 10.1097/MAJ.0b013e318235597b, PMID: 22157388

[29] Alcaide ML Rodriguez VJ Brown MR Pallikkuth S Arheart K Martinez O. High levels of inflammatory cytokines in the reproductive tract of women with BV and engaging in intravaginal douching: a cross-sectional study of participants in the women interagency HIV study. AIDS Res Hum Retroviruses. (2017) 33:309–17. doi: 10.1089/aid.2016.0187, PMID: 27897054 PMC5372759

[30] Hedges SR Barrientes F Desmond RA Schwebke JR. Local and systemic cytokine levels in relation to changes in vaginal flora. J Infect Dis. (2006) 193:556–62. doi: 10.1086/499824, PMID: 16425135

[31] Lopez-Castejon G Brough D. Understanding the mechanism of IL-1beta secretion. Cytokine Growth Factor Rev. (2011) 22:189–95. doi: 10.1016/j.cytogfr.2011.10.001, PMID: 22019906 PMC3714593

[32] Sha BE Chen HY Wang QJ Zariffard MR Cohen MH Spear GT. Utility of Amsel criteria, Nugent score, and quantitative PCR for *Gardnerella vaginalis*, mycoplasma hominis, and *Lactobacillus* spp. for diagnosis of bacterial vaginosis in human immunodeficiency virus-infected women. J Clin Microbiol. (2005) 43:4607–12. doi: 10.1128/JCM.43.9.4607-4612.2005, PMID: 16145114 PMC1234056

[33] Martino JL Vermund SH. Vaginal douching: evidence for risks or benefits to women's health. Epidemiol Rev. (2002) 24:109–24. doi: 10.1093/epirev/mxf004, PMID: 12762087 PMC2567125

[34] Sierra LJ Brown AG Barila GO Anton L Barnum CE Shetye SS. Colonization of the cervicovaginal space with *Gardnerella vaginalis* leads to local inflammation and cervical remodeling in pregnant mice. PLoS One. (2018) 13:e0191524. doi: 10.1371/journal.pone.0191524, PMID: 29346438 PMC5773211

[35] Kim JY Moon EC Kim JY Kim HJ Heo K Shim JJ. *Lactobacillus helveticus* HY7801 ameliorates bacterial vaginosis by inhibiting biofilm formation and epithelial cell adhesion of *Gardnerella vaginalis*. Food Sci Biotechnol. (2022) 32:507–15. doi: 10.1007/s10068-022-01208-7 36911333 PMC9992491

[36] Teixeira GS Carvalho FP Arantes RME Nunes AC Moreira JLS Mendonça M. Characteristics of Lactobacillus and *Gardnerella vaginalis* from women with or without bacterial vaginosis and their relationships in gnotobiotic mice. J Med Microbiol. (2012) 61:1074–81. doi: 10.1099/jmm.0.041962-0, PMID: 22539000

[37] Laniewski P Herbst-Kralovetz MM. Bacterial vaginosis and health-associated bacteria modulate the immunometabolic landscape in 3D model of human cervix. NPJ Biofilms Microbiomes. (2021) 7:88. doi: 10.1038/s41522-021-00259-8, PMID: 34903740 PMC8669023

[38] Gillet E Meys JFA Verstraelen H Bosire C De Sutter P Temmerman M. Bacterial vaginosis is associated with uterine cervical human papillomavirus infection: a meta-analysis. BMC Infect Dis. (2011) 11:10. doi: 10.1186/1471-2334-11-10, PMID: 21223574 PMC3023697

[39] Ding CF Yu YS Zhou Q. Bacterial vaginosis: effects on reproduction and its therapeutics. J Gynecol Obstet Hum Reprod. (2021) 50:102174. doi: 10.1016/j.jogoh.2021.102174 34087449

[40] Gilbert NM Lewis WG Lewis AL. Clinical features of bacterial vaginosis in a murine model of vaginal infection with *Gardnerella vaginalis*. PLoS One. (2013) 8:e59539. doi: 10.1371/journal.pone.0059539, PMID: 23527214 PMC3602284

[41] Kristiansen FV Oster S Frost L Boustouller Y Korsager B Moller BR. Isolation of *Gardnerella vaginalis* in pure culture from the uterine cavity of patients with irregular bleedings. Br J Obstet Gynaecol. (1987) 94:979–84. doi: 10.1111/j.1471-0528.1987.tb02273.x, PMID: 3500741

[42] Moon EC Park MS Lim T Kim RH Ji GE Kim SY. Antibacterial effect of cell-free supernatant fraction from *Lactobacillus paracasei* CH88 against *Gardnerella vaginalis*. Sci Rep. (2022) 12:4763. doi: 10.1038/s41598-022-08808-7, PMID: 35306534 PMC8934363

