# Supplementary figures and images for: Development of the standard mouse model for human bacterial vaginosis induced by Gardnerella vaginalis
Source: Front Vet Sci. 2023 Sep 12;10:1226859. doi: 10.3389/fvets.2023.1226859 (PMC10536170; doi:10.3389/fvets.2023.1226859)

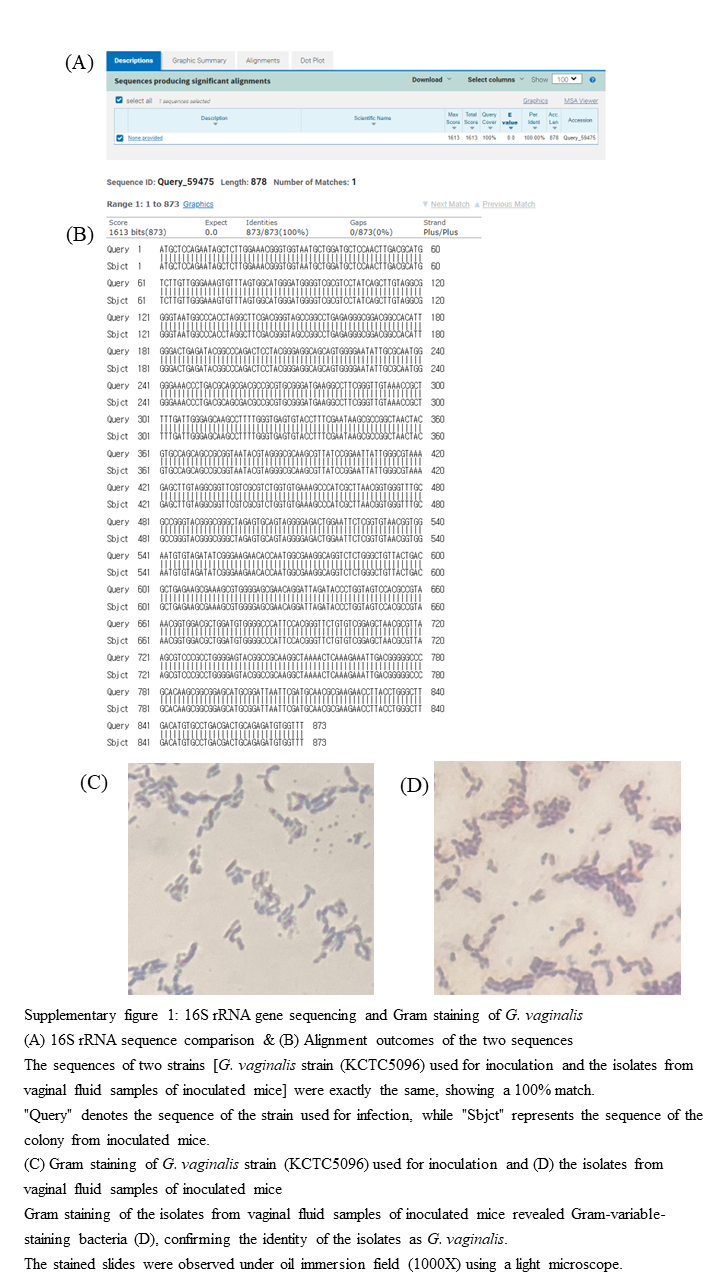

Supplement: Supplementary file 1 [file Image_1.tif]
